# 肺腺样囊性癌4例病例报告

**DOI:** 10.3779/j.issn.1009-3419.2017.11.12

**Published:** 2017-11-20

**Authors:** 玺兰 贺, 建华 陈

**Affiliations:** 410000 长沙，中南大学湘雅医学院，中南大学湘雅医学院附属肿瘤医院胸部内一科 Xiangya School of Medicine Central South University, Department of Thoracic Neoplasms, Hunan Cancer Hospital, the Affliated Cancer Hospital of Xiangya School of Medicine, Changsha 410000, China

**Keywords:** 肺腺样囊性癌, 肺肿瘤, 诊断, 治疗, Lung adenoid cystic carcinoma, Lung neoplasms, Diagnosis, Treatment

## Abstract

**背景与目的:**

肺腺样囊性癌是肺癌中的少见类型，通常对其诊断及治疗认识不足。为了提高对肺腺样囊性癌的认识，本文对该病例进行了收集和分析。

**方法:**

回顾分析我院2012年1月-2016年12月收治的4例肺腺样囊性癌，对其病理免疫组化、表皮生长因子受体（epidermal growth factor receptor, *EGFR*）及间变性淋巴瘤激酶（anaplastic lymphoma kinase, *ALK*）突变分析、诊断和治疗特点进行总结。

**结果:**

肺腺样囊性癌是一种主要发生在气道、*EGFR*及*ALK*突变少见、转移较晚、手术治疗效果好的一种疾病。

**结论:**

肺腺样囊性癌诊断主要依赖于病理学，早期以手术治疗为主，晚期可行放化疗，但靶向治疗机会不多，其预后较小细胞肺癌及非小细胞肺癌好。

## Abstract (en)

**Background and objective:**

Lung adenoid cystic carcinoma is a kind of rare lung cancer. Diagnosis and treatment is not enough understandable for them. We collected and analyzed 4 cases of lung adenoid cystic carcinoma for broadening the sight of this disease.

**Methods:**

Retrospectively analysed the 4 cases we collected from Hunan Cancer Hospital Between January 2012 and December 2016. We depicted the pathology, immunohistochemical, epidermal growth factor receptor (*EGFR*) mutation and anaplastic lymphoma kinase (*ALK*) arrangement in these cases. And the methods of the diagnosis and treatment were analyzed.

**Results:**

Lung adenoid cystic carcinoma is usually located in the airway, *EGFR* mutation and *ALK* arrangement is rare in this disease. Generally the metastasis of the lung cancer occurred in the advanced stage. The prognosis is good if the mass could be resected completely.

**Conclusion:**

Diagnosis of the lung adenoid cystic carcinoma depends on pathological experiments, surgery is the main treatment in the early stage, radiotherapy and chemotherapy is an advisable therapy in the advanced stage. And the prognosis of this kind of lung cancer is better than small cell lung cancer and non-small cell lung cancer.

肺癌是我国非常常见的肺部肿瘤，不同的病理学分型及分期的肺癌患者，其治疗方案也千差万别。肺腺样囊性癌是涎腺样中肿瘤的一种，发病率极低，在临床极为少见。本文收集了2012年1月-2016年12月间我院收诊的4例肺腺样囊性癌患者，通过对患者病理学分型，免疫组化检查，表皮生长因子受体（epidermal growth factor receptor, *EGFR*）及间变性淋巴瘤激酶（anaplastic lymphoma kinase, *ALK*）突变检测，影像学表现，治疗及预后情况进行汇报，分析该疾病的治疗及预后情况。

## 1 病例资料

### 1.1 病例1

患者男，78岁，吸烟30余年，20支每天。因“咳嗽1个月，胸痛10天”2014年在当地医院行胸部计算机断层扫描（computed tomography, CT）检查，检查提示：“右下肺病变性质待查：肺癌？结核瘤？”患者为求进一步诊疗，遂来我院。肺部增强CT（图 1A、图 1B）：1.右下肺肿块，性质待定，肺癌？炎性假瘤？并右下肺炎性病变。2.慢性支气管疾患，双肺肺大泡及少许纤维化灶。B超：肝、胆、脾脏、胰腺、双肾未见明显异常；腹腔、腹膜后、双侧肾上腺区未见明显肿块；心内结构未见明显异常；左心舒张功能减退、收缩功能正常范围。脑磁共振成像（magnetic resonance imaging, MRI）平扫+增强：双侧基底节及放射冠区多发腔隙性梗塞灶；脑白质疏松。完善支气管镜检查：提示支气管炎。考虑肿块局限，遂于2014年9月22日予以胸腔镜手术治疗。术后病理结果：腺样囊性癌。免疫组化：CD117（++）、LCK（+）、CK-7（散在个别+）、GCDFP-15（-）、HCK（++）、CK-P（+）、CD56（-）、CD99（-）、EMA（-）、VIM（++）、TTF-1（-）、P63（-）、SYN（-）、CGA（-）、CK5/6（++）。完善肿瘤组织EGFR突变检测：19号外显子与21号外显子均无突变。ALK突变检测（-）。术后患者未行其他治疗，随访至今，患者目前仍健在。

**1:**
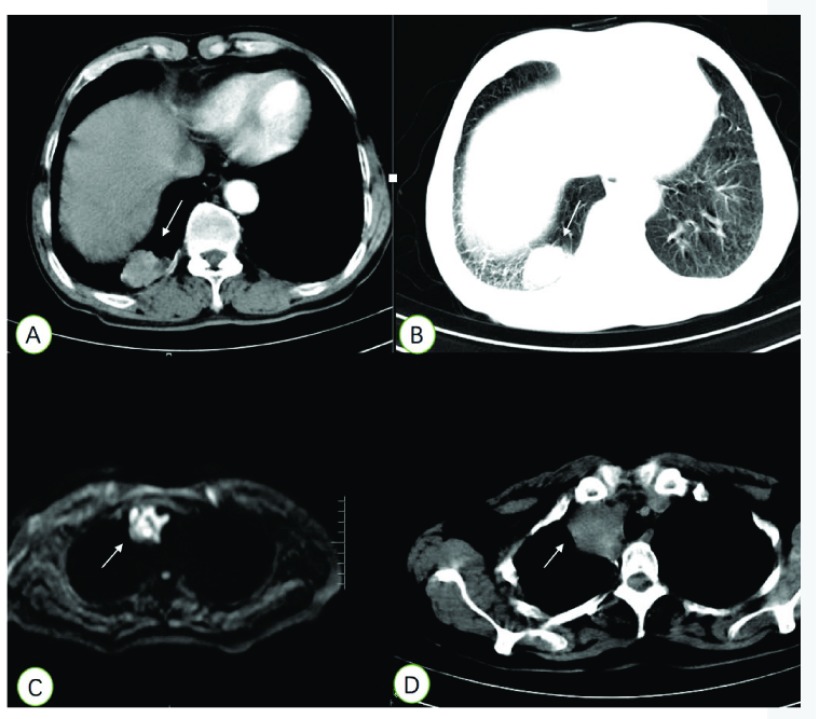
病例影像学资料：A、B为病例1患者肺部CT检查资料，箭头处可见该肿块，该肿块位于右下叶后基底段；C、D为病例2患者肺部及纵隔MRI及CT纵膈窗检查资料，箭头处可见该肿块。 Imaging data of cases. A and B belong to Case 1, the mass could be seen from arrow, the mass was located in the lower lobe basal segment; C, D belong to Case 2, figure C was MRI data in lung and mediastinumand, D was CT scan data of this mass. MRI: magnetic resonance imaging; CT: computed tomography.

### 1.2 病例2

患者女，50岁。无吸烟史。2013年11月发现“右肺肿块”，行支气管镜检查提示：右肺主气管可见粘膜肥厚肿胀，粗糙不平，管腔内狭窄堵塞。行外科手术，术后病检提示：右下肺中分化腺样囊性癌，肿块最大径2.8 cm，支气管残端未见癌累及，送检第7、9、11、12组淋巴结未见癌转移。2016年7月因受凉后出现头晕、咳嗽，无明显发热、胸闷等不适。在当地医院予以抗炎治疗。未见明显好转，2016年8月复查胸部CT发现右前上纵隔肿块。遂再次来我院，体查发现颈部淋巴结肿大，肺部CT及胸部MRI（图 1C、图 1D）提示纵隔淋巴结肿大。完善颈部淋巴结活检，考虑肺腺样囊性癌淋巴结转移。完善血液*EGFR*突变检测，检测提示：19号外显因子与21号外显因子均无突变。予以行长春瑞滨74 mg+顺铂100 mg方案化疗，3周期后患者纵隔淋巴结较前增大，考虑疾病进展，后改放疗100% GTVnd 60 Gy/28次+100% CTVnd 60 Gy/28次+98.2% PTVnd 60 Gy/28次+紫杉醇脂质体120 mg单药化疗，后续仍用长春瑞滨74 mg+顺铂100 mg方案化疗3周期及贝伐珠单抗400 mg抗肿瘤血管生成。病情稳定后持续使用贝伐珠单抗抗血管生成维持治疗。随访至今患者颈部及纵隔淋巴结未见继续增大，仍在用贝伐珠单抗抗肿瘤血管生成。

### 1.3 病例3

患者男，62岁，因“刺激性咳嗽10天，发现左肺肿块1周”于2012年入院，完善检查后于2012年10月12日行手术治疗，术中病理检查提示：肺腺样囊性癌。肿瘤组织EGFR突变检测：19号外显子与21号外显子均无突变。术后每年复查肺部CT，2017年电话随访患者，患者目前良好，2017年4月复查肺部CT未见肿块。未服用药物。

### 1.4 病例4

患者男，70岁，因“声嘶18天”于2014年入住我院，完善肺部+腹部增强CT，提示：左上肺肿块，考虑肺癌可能性大，伴双侧肾上腺及腹膜后转移。支气管镜检查：发现左上叶管腔见新生物阻塞。活检行病理学检查提示：实性腺样囊性癌，免疫组化结果提示：免疫组化：Ki-67约20%、CK5/6（-）、P63（-）、SYN（-）、TTF-1（-）、CGA（-）、LCK（-）、HCK（+）、CD117（+）、PLAP（-）。*EGFR*及*ALK*突变检测均（-）。行肺癌综合讨论，考虑患者目前病变范围广，不考虑行手术及放疗，遂予以行紫杉醇脂质体240 mg及卡铂400 mg方案化疗1周，后患者拒绝化疗予以出院，后患者6个月后死亡。

## 2 讨论

腺样囊性癌（adenoid cystic carcinoma, ACC）是涎腺肿瘤^[1]^，可出现远处转移。腺样囊性癌常发生在头颈部唾液腺，腺样囊性癌也少见发生在脾脏、肾脏、骨、淋巴结、肝脏^[2]^。然而原发于肺部的腺样囊性癌更少见，占肺癌患者的0.09%-0.2%^[3]^。既往认为这是一种低度恶性肿瘤，并且患者较常见的肺癌类型如非小细胞肺癌患者预后好。该病主要发病于50岁-60岁，男女发病率无性别差异，发病率及预后与是否吸烟无关。本文4例患者中2例未吸烟。

大多数肺腺样囊性癌发生于大气道，也可发生于小气道。本文4例患者中3例发生于大气道，1例发生在小气道。即使该肿瘤被认为是低度恶性肿瘤，但晚期患者可出现全身转移及肿块压迫气管导致使患者窒息死亡。本文只有1例患者病情晚，发现时已转移至腹膜后淋巴结，不能手术仅行内科治疗，预后不好，其他3例均可手术治疗，2例治愈，1例复发，复发后患者经放化疗，治疗效果尚好，维持治疗存活中。

目前研究认为：肺腺样囊性癌病程长，从出现肺部肿块到患者出现症状可达数年至数十年。早期发现手术治疗多数可治愈。该肿瘤也可出现远处转移，一般是原发肿瘤出现数年后再发生远处转移，可通过淋巴道转移至纵隔淋巴结，腹膜后淋巴结；也可通过血道转移至肝、骨、心脏瓣膜、肾上腺等^[4, 5]^。本研究结果与之相符。

该肿瘤病理学一般表现有3种组织学亚型，即实性、囊状和管状，但分级困难，因为肿瘤可能会显示多于一种亚型的病理学表现。患者病理学表现提示为实性，可能预后更差。但是目前认为对于该疾病预后分析，分期比病理及免疫组化都更为重要。细胞学研究发现瘤体内存在重复的中型大小的肿瘤细胞聚集。该瘤体细胞核浓染颗粒，均匀分布的染色质。肿瘤细胞通常围绕一个核心安排均匀黏液样物质，或三维形式及“球形”集群^[6]^。目前研究^[7]^发现，miR-205在原发腺样囊性癌中高表达，然而miR-155和miR-342在复发的腺样囊性癌中高表达。免疫组化检查可出现：细胞角蛋白（CK）、p63、S-100、波形蛋白（vimentin）和平滑肌肌动蛋白表达阳性。而甲状腺转录因子-1、突触小泡蛋白、CD-56、CK20及嗜铬粒蛋白A不表达^[8]^。

针对肺腺样囊性癌患者行基因突变检测，本文报告该4例患者均未出现*EGFR*突变阳性，2例行肿瘤组织*ALK*突变检测均提示（-）。Huo等^[9]^在24例原发肺腺样囊性癌患者通过桑格测序法、二代基因检测等方法行*EGFR*、*KRAS*、*BRAF*、*ALK*、*PIK3CA*、*PDGFRA*和*DDR2*突变检测，24例患者均未发现基因突变检测。有文献^[3]^报道对32例肺腺样囊性癌患者肿瘤组织行*KIT*基因突变检测，32例均表达阳性，且其中18例患者提示所测肿瘤组织中50%出现KIT表达检测阳性。且小型临床试验提示该类肿瘤对靶向药物不敏感^[10]^，考虑该类肿瘤*EGFR*、*ALK*突变可能低，针对*EGFR*及*ALK*突变的靶向药物治疗不理想。然而也有个例报道肺腺样囊性癌存在*EGFR*突变，2016年日本报道了1例80岁老年女性肺腺样囊性癌患者病理检测出现*EGFR*突变，患者每日口服吉非替尼250 mg，病灶缩小。提示肺腺样囊性癌极少存在*EGFR*突变，偶有*EGFR*突变，口服EGFR-TKI有效^[11]^。本文4例患者均无*EGFR*突变，2例无*ALK*突变，与多数文献报道相符。

早期肺腺样囊性癌主要以手术治疗为主，但由于影像学无法准确评估该肿块大小及范围，且腺样囊性癌倾向于浸润神经，导致大约30%手术患者可出现切缘阳性，出现术后复发转移。在我院就诊的4例肺腺样囊性癌患者中3例行手术治疗，2例治愈，1例复发，1例出现多处转移，分期较晚患者预后明显不良。

肺腺样囊性癌是一种放疗敏感性肿瘤^[12]^。对于无法切除的肺腺样囊性癌，手术病理提示切缘阳性，以及术后原位复发无法再次行手术的患者，放疗是可行的治疗方式，对于能够完全切除且切缘阴性的肺腺样囊性癌，是否行术后放疗，目前文献存在争议^[13]^。

对于晚期无法行手术及放疗的患者，且患者肿块出现快速进展增大及压迫症状时可考虑化疗^[14]^。目前已有5-氟尿嘧啶、顺铂、表柔比星、紫杉醇、吉西他滨单药化疗对于治疗反应的评估。然而仅在顺铂及米托蒽醌组少数患者出现完全缓解，其余组均未见完全缓解。目前常用化疗是含铂的第三代化疗药物联合的双药方案。

随着分子靶向药物的发展，对于晚期无法行手术、放疗及化疗患者，分子靶向药物临床试验也在进行。目前已有伊马替尼^[15]^、吉非替尼^[16]^、硼替佐米^[17]^、拉帕替尼^[10]^、西妥昔单抗^[18]^的小型临床试验结果，然而目前临床实验结果均不甚理想，暂未予以加入临床推荐治疗。

目前暂未见免疫治疗及CAR-T治疗用于肺腺样囊性癌临床试验，但是对于晚期肺腺样囊性癌患者我们期待更多更好的临床治疗。

肺腺样囊性癌预后，对于能行完全切除腺样囊性癌患者其5年生存率为100%，其10年生存率为90%^[19]^。对于不能行完全切除的腺样囊性癌生存期降低，5年及10年生存率仅为33.3%-53%^[20]^。

总之，肺腺样囊性癌手术患者预后较好，正确认识，早期发现能提高患者生存率，但晚期预后不好。虽然该肺部肿瘤发病率低，但在临床实践中仍可遇见，其发生机制与常见的小细胞肺癌及非小细胞肺癌有区别，预后也存在差异。
